# Prevalence of Arthritis and Arthritis-Attributable Activity Limitation — United States, 2016–2018

**DOI:** 10.15585/mmwr.mm7040a2

**Published:** 2021-10-08

**Authors:** Kristina A. Theis, Louise B. Murphy, Dana Guglielmo, Michael A. Boring, Catherine A. Okoro, Lindsey M. Duca, Charles G. Helmick

**Affiliations:** ^1^Division of Population Health, National Center for Chronic Disease Prevention and Health Promotion, CDC; ^2^Oak Ridge Institute for Science and Education, Oak Ridge, Tennessee; ^3^ASRT Inc., Smyrna, Georgia; ^4^Division of Human Development and Disability, National Center on Birth Defects and Developmental Disabilities, CDC; ^5^Epidemic Intelligence Service, CDC.

Arthritis has been the most frequently reported main cause of disability among U.S. adults for >15 years (*1*), was responsible for >$300 billion in arthritis-attributable direct and indirect annual costs in the U.S. during 2013 (*2*), is linked to disproportionately high levels of anxiety and depression (*3*), and is projected to increase 49% in prevalence from 2010-2012 to 2040 (*4*). To update national prevalence estimates for arthritis and arthritis-attributable activity limitation (AAAL) among U.S. adults, CDC analyzed combined National Health Interview Survey (NHIS) data from 2016–2018. An estimated 58.5 million adults aged ≥18 years (23.7%) reported arthritis; 25.7 million (10.4% overall; 43.9% among those with arthritis) reported AAAL. Prevalence of both arthritis and AAAL was highest among adults with physical limitations, few economic opportunities, and poor overall health. Arthritis was reported by more than one half of respondents aged ≥65 years (50.4%), adults who were unable to work or disabled* (52.3%), or adults with fair/poor self-rated health (51.2%), joint symptoms in the past 30 days (52.2%), activities of daily living (ADL)^†^ disability (54.8%), or instrumental activities of daily living (IADL)^§^ disability (55.9%). More widespread dissemination of existing, evidence-based, community-delivered interventions, along with clinical coordination and attention to social determinants of health (e.g., improved social, economic, and mental health opportunities), can help reduce widespread arthritis prevalence and its adverse effects.

NHIS is an ongoing, nationally representative, in-person interview health survey of the noninstitutionalized, U.S. civilian population. Analyses were limited to adults aged ≥18 years. Unweighted sample sizes and final response rates of the Sample Adult component^¶^ for 2016, 2017, and 2018 were 33,028 (54.3%); 26,742 (53.0%); and 25,417 (53.1%), respectively. Arthritis was ascertained by a response of “yes” to, “Have you ever been told by a doctor or other health care professional that you have arthritis, rheumatoid arthritis, gout, lupus, or fibromyalgia?” AAAL was ascertained among those with arthritis by a response of “yes” to, “Are you now limited in any way in any of your usual activities because of arthritis or joint symptoms?” Annualized unadjusted and age-standardized** prevalence estimates of arthritis and AAAL were generated overall and by selected sociodemographic,^††^ health,^§§^ and function characteristics.^¶¶^ Sampling weights were applied to account for the complex survey design, to generate nationally representative estimates, and to adjust for nonresponse. Subgroup differences were assessed using pairwise t-tests; orthogonal linear contrasts were performed to conduct linear trend tests in ordinal variables. Unadjusted estimates are reported in text unless otherwise noted; all differences are significant at α = 0.05. To examine change over time, a secondary analysis using identical methods was conducted to produce annualized absolute prevalence estimates of arthritis and AAAL for the combined years 2003–2005, 2007–2009, 2010–2012, and 2013–2015. These years were chosen to correspond to previous surveillance reports.*** A linear model trend test was conducted with significance set at α = 0.05.^†††^ Analyses were conducted in SAS (version 9.4; SAS Institute) and SUDAAN (version 11.0; RTI International). This activity was reviewed by CDC and was conducted consistent with applicable federal law and CDC policy.^§§§ ^

During 2016–2018, 58.5 million U.S. adults aged ≥18 years (23.7%; 21.5% age-standardized) are estimated to have arthritis; 25.7 million (43.9%; 40.8% age-standardized) of those with arthritis are estimated to have AAAL (Figure), representing 10.4% (9.4% age-standardized) of the total U.S. adult population. Annualized absolute prevalence of both arthritis and AAAL continues nearly two decades of an increasing statistically significant linear trend (Figure). Prevalence of arthritis increased with increasing age, body mass index (BMI), aerobic physical inactivity, and worsening psychological distress and self-rated health, and decreased with increasing educational attainment and income-to-poverty ratio (Table 1). Arthritis prevalence was >50% among adults aged ≥65 years (50.4%), adults who were unable to work or disabled (52.3%), and adults with fair/poor self-rated health (51.2%), joint symptoms in the past 30 days (52.2%), ADL disability (54.8%), and IADL disability (55.9%).

**FIGURE:**
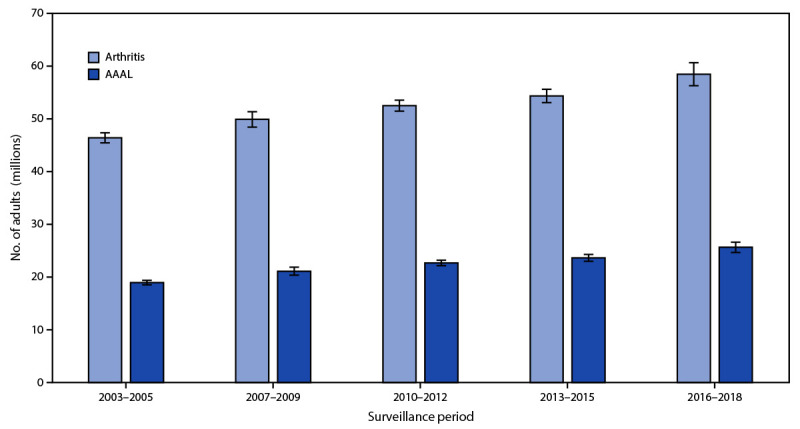
Weighted number of adults aged ≥18 years with arthritis* and arthritis-attributable activity limitation^†^^,^^§^^,^^¶^^,^** — National Health Interview Survey, United States, 2003–2018 **Abbreviation**: AAAL = arthritis-attributable activity limitation. * Responded “yes” to, “Have you ever been told by a doctor or other health professional that you have arthritis, rheumatoid arthritis, gout, lupus, or fibromyalgia?” ^†^ Responded “yes” to, “Are you now limited in any way in any of your usual activities because of arthritis or joint symptoms?” ^§^ 95% confidence intervals indicated by error bars. ^¶^ Separate linear model trend tests were conducted for both outcomes with significance set at α = 0.05. ** The p for trend for both outcomes was <0.001.

**TABLE 1:** Unadjusted and age-standardized* annualized prevalence of doctor-diagnosed arthritis^†^ among adults aged ≥18 years — National Health Interview Survey, United States, 2016–2018

Characteristic	No. of adults with arthritis (unweighted)^§^	No. of adults with arthritis in population^§^ (millions)	Distribution among adults with arthritis^¶^ (%)	Prevalence of doctor-diagnosed arthritis, % (95% CI)	
				Unadjusted	Age-standardized
**Overall**	**23,921**	**58.5**	**100**	**23.7 (23.2–24.2)**	**21.5 (21.1–21.9)**
**Sociodemographic characteristic**					
**Age group, yrs**					
18–44	2,594	8.1	13.8	7.1 (6.7–7.5)	—
45–64	9,313	25.4	43.5	30.5 (29.7–31.4)	—
≥65	12,014	25.0	42.7	50.4 (49.4–51.3)	—
**Sex**					
Male	9,159	23.8	40.7	20.0 (19.4–20.6)	18.5 (18.0–19.0)
Female	14,762	34.7	59.3	27.2 (26.5–27.8)	24.2 (23.6–24.7)
**Race/Ethnicity**					
Hispanic or Latino, any race	1,733	5.4	9.3	13.7 (12.7–14.6)	16.4 (15.5–17.3)
White, NH	18,399	43.4	74.2	27.6 (27.0–28.2)	23.2 (22.7–23.7)
Black, NH	2,548	6.4	10.9	22.0 (20.8–23.2)	21.8 (20.8–22.9)
Asian or Other Pacific Islander, NH	549	1.7	3.0	11.9 (10.6–13.2)	12.2 (11.0–13.5)
American Indian or Alaska Native, NH	211	0.5	0.8	26.3 (20.9–31.6)	26.8 (22.0–32.3)
Other/Multiple races, NH	481	1.1	1.9	23.5 (20.9–26.1)	26.9 (24.6–29.4)
**Sexual identity**					
Lesbian or gay	356	0.8	1.4	21.2 (18.9–23.6)	22.4 (20.0–25.0)
Straight	22,498	55.1	96.5	23.9 (23.3–24.4)	21.4 (21.0–21.9)
Bisexual	197	0.5	0.8	16.9 (14.1–19.8)	25.6 (22.1–29.5)
Something else/Don't know the answer**	336	0.7	1.2	22.2 (19.1–25.5)	22.4 (19.8–25.2)
**Education**					
Less than high school graduate	3,310	7.8	13.4	26.9 (25.7–28.0)	22.0 (21.1–23.0)
High school graduate or equivalent	6,494	16.0	27.4	26.5 (25.5–27.4)	23.0 (22.2–23.7)
Some college	7,631	18.3	31.4	24.3 (23.5–25.1)	23.4 (22.7–24.0)
College degree or greater	6,410	16.2	27.8	20.0 (19.3–20.7)	18.5 (18.0–19.1)
**Employment status**					
Employed/Self-employed	8,849	24.4	41.8	15.7 (15.2–16.2)	18.2 (17.7–18.8)
Unemployed	475	1.3	2.2	14.9 (13.3–16.5)	20.0 (17.9–22.2)
Unable to work/Disabled^††^	3,578	8.4	14.4	52.3 (50.6–54.0)	43.1 (41.3–44.9)
Other**^§§^**	11,012	24.3	41.7	36.7 (35.7–37.7)	21.8 (21.1–22.6)
**Income-to-poverty ratio^¶¶^**					
Poor/Near poor (<125%)	4,811	10.1	17.2	24.7 (23.7–25.8)	25.7 (24.8–26.7)
Low income (125% to <200%)	3,554	7.9	13.6	25.1 (24.0–26.1)	22.7 (21.8–23.7)
Middle income (200% to <400%)	6,972	17.1	29.2	24.1 (23.3–24.9)	21.6 (21.0–22.3)
High income (≥400%)	8,583	23.4	40.0	22.6 (22.0–23.3)	19.7 (19.2–20.3)
**Health characteristic**					
**BMI (kg/m^2^)**					
Under/Healthy weight (<25.0)	6,128	14.6	25.8	17.6 (17.0–18.2)	17.2 (16.7–17.7)
Overweight (25.0 to <30.0)	7,609	18.4	32.6	22.2 (21.6–22.9)	19.1 (18.5–19.6)
Obese (≥30.0)	9,349	23.5	41.6	32.0 (31.1–32.9)	28.8 (28.1–29.5)
Aerobic physical activity level***					
Meets recommendations	9,598	24.1	41.9	18.8 (18.3–19.4)	19.1 (18.6–19.6)
Insufficiently active	5,443	13.3	23.1	27.0 (26.1–27.9)	23.4 (22.7–24.2)
Inactive	8,426	20.1	34.9	30.9 (29.9–31.9)	24.3 (23.5–25.1)
**Joint symptoms^†††^**					
Yes	17,973	43.9	75.1	52.2 (51.4–53.0)	42.0 (41.1–42.8)
No	5,943	14.6	24.9	9.0 (8.6–9.3)	9.6 (9.3–10.0)
**ADL disability^§§§^**					
Yes	1,493	3.3	5.7	54.8 (52.2–57.5)	41.4 (37.9–44.9)
No	22,426	55.2	94.3	22.9 (22.4–23.4)	21.1 (20.6–21.5)
**IADL disability^¶¶¶^**					
Yes	3,098	6.5	11.1	55.9 (54.0–57.7)	41.8 (39.5–44.0)
No	20,818	52.0	88.9	22.1 (21.6–22.6)	20.5 (20.1–21.0)
Psychological distress****					
None/Mild	16,450	40.2	70.7	21.4 (20.8–21.9)	18.8 (18.4–19.3)
Moderate	5,236	12.8	22.5	29.9 (28.9–30.8)	29.4 (28.6–30.3)
Serious	1,589	3.9	6.8	44.3 (42.0–46.7)	41.1 (39.1–43.2)
**Self-rated health**					
Excellent/Very good	9,198	22.8	38.9	15.2 (14.7–15.7)	15.8 (15.4–16.3)
Good	8,027	19.6	33.5	29.9 (29.0–30.8)	25.0 (24.2–25.8)
Fair/Poor	6,684	16.1	27.6	51.2 (49.8–52.5)	40.7 (39.2–42.2)

**Abbreviations**: ADL = activities of daily Living; BMI = body mass index; CI = confidence interval; IADL = instrumental activities of daily living; NH = non-Hispanic.

* Age-standardized to the 2000 U.S. projected adult population, using three age groups: 18–44, 45–64, and ≥65 years.

^†^ Responded “yes” to, “Have you ever been told by a doctor or other health professional that you have some form of arthritis, rheumatoid arthritis, gout, lupus, or fibromyalgia?”

^§^ Might not sum to overall total for some categories because of item-specific missing data.

^¶^ Might not sum to 100 because of rounding.

** Responded “I don't know the answer” to, “Which of the following best represents how you think of yourself?”

^††^ This category is a combination of respondents who self-reported their reason for not working as: “temporarily unable to work due to health reasons” or “disabled.”

^§§^ Students, homemakers, and retirees.

^¶¶^ Income-to-poverty ratio estimates were derived using NHIS imputed income file. https://www.cdc.gov/nchs/data/nhis/tecdoc18.pdf

*** Respondents were considered to have met recommendations if they reported ≥150 minutes of moderate-intensity leisure-time aerobic physical activity per week, insufficiently active if they reported 1–149 minutes, and inactive if they reported 0 minutes. Reported vigorous-intensity physical activity minutes were counted twice and added to moderate-intensity physical activity minutes.

^†††^ Responded “yes” to, “The next questions refer to your joints. Please do not include the back or neck. During the past 30 days, have you had any symptoms of pain, aching, or stiffness in or around a joint?”

^§§§^ Responded “yes” to, “Because of a physical, mental, or emotional problem, [do you] need the help of other persons with personal care needs, such as eating, bathing, dressing, or getting around inside this home?”

^¶¶¶^ Responded “yes” to, “Because of a physical, mental, or emotional problem, [do you] need the help of other persons in handling routine needs, such as everyday household chores, doing necessary business, shopping, or getting around for other purposes?”

**** Psychological distress was classified as none/mild, moderate, or severe and measured by the Kessler-6 Scale. https://www.hcp.med.harvard.edu/ncs/k6_scales.php

Among adults with arthritis, unadjusted prevalence of AAAL exceeded 50% in several groups, including adults with joint symptoms in the past 30 days (51.6%), adults who were unable to work or disabled (54.7%), adults of other/multiple races (54.5%) or non-Hispanic American Indian or Alaska Natives (60.7%), adults with low income (53.3%) or poor/near poor income-to-poverty ratios (63.3%), or with moderate psychological distress (59.5%) (Table 2). AAAL was also reported by a high proportion of adults with arthritis who had an ADL disability (82.6%), IADL disability (80.4%), serious psychological distress (76.3%), or fair/poor self-rated health (72.6%).

**TABLE 2:** Unadjusted and age-standardized* annualized prevalence of arthritis-attributable activity limitation^†^ among adults aged ≥18 years and unadjusted and age-standardized prevalence of arthritis-attributable activity limitation among those with doctor-diagnosed arthritis^§^ — National Health Interview Survey, United States, 2016–2018

Characteristic	Unweighted no. of adults with arthritis^¶^	No. of adults with AAAL in population^¶^ (millions)	Distribution among adults with AAAL** (%)	Prevalence of AAAL among all US adults, % (95% CI)		Prevalence of AAAL among adults with doctor-diagnosed arthritis, % (95% CI)	
				Unadjusted	Age-standardized	Unadjusted	Age-standardized
**Overall**	**10,682**	**25.7**	**100**	**10.4 (10.1–10.7)**	**9.4 (9.1–9.6)**	**43.9 (42.9–44.8)**	**40.8 (39.4–42.1)**
**Sociodemographic characteristic**							
**Age group, yrs**							
18–44	996	3.0	11.6	2.6 (2.4–2.8)	N/A	36.8 (34.6–39.1)	N/A
45–64	4,378	11.7	45.6	14.0 (13.5–14.6)	N/A	46.0 (44.5–47.5)	N/A
≥65	5,308	11.0	42.8	22.2 (21.5–22.9)	N/A	44.0 (42.9–45.2)	N/A
**Sex**							
Male	3,831	9.6	37.6	8.1 (7.8–8.5)	7.5 (7.2–7.8)	40.6 (39.3–41.9)	37.1 (35.1–39.3)
Female	6,851	16.0	62.4	12.5 (12.1–13.0)	11.1 (10.7–11.5)	46.1 (45.0–47.3)	43.3 (41.5–45.1)
**Race/Ethnicity**							
Hispanic or Latino, any race	875	2.7	10.4	6.7 (6.1–7.4)	8.2 (7.5–8.9)	49.1 (46.0–52.3)	43.4 (39.3–47.6)
White, NH	7,854	18.2	71.0	11.6 (11.2–12.0)	9.6 (9.3–9.9)	41.9 (40.9–43.0)	39.3 (37.7–40.9)
Black, NH	1,300	3.1	12.2	10.8 (9.9–11.7)	10.6 (9.9–11.5)	48.9 (46.2–51.6)	43.2 (39.3–47.1)
API, NH	244	0.8	3.1	5.5 (4.6–6.5)	5.7 (4.8–6.6)	46.2 (40.4–52.1)	42.8 (34.0–52.1)
AI/AN, NH	134	0.3	1.1	15.9 (12.9–19.6)	16.3 (13.5–19.6)	60.7 (50.3–70.2)	58.9 (46.0–70.8)
Other/Multiple races, NH	275	0.6	2.3	13.1 (11.0–15.5)	15.1 (13.0–17.5)	54.5 (48.5–60.3)	54.2 (46.4–61.7)
**Sexual identity**							
Lesbian or gay	163	0.4	1.6	10.2 (8.3–12.1)	10.5 (8.7–12.7)	48.1 (41.3–54.8)	47.8 (39.6–56.1)
Straight	9,960	24.0	96.0	10.4 (10.0–10.7)	9.3 (9.0–9.5)	43.5 (42.5–44.5)	40.1 (38.7–41.6)
Bisexual	103	0.2	1.0	8.7 (6.5–11.0)	13.1 (10.2–16.6)	51.7 (42.8–60.6)	50.8 (42.2–59.4)
Something else/Don't know the answer^††^	169	0.3	1.4	10.7 (8.8–13.0)	11.0 (9.2–13.1)	48.4 (41.2–55.8)	51.5 (41.0–61.8)
**Education**							
Less than HS graduate	1,902	4.5	17.5	15.4 (14.5–16.3)	12.4 (11.8–13.2)	57.2 (55.0–59.3)	51.1 (46.9–55.2)
HS graduate or equivalent	2,954	7.1	27.9	11.8 (11.2–12.3)	10.2 (9.7–10.7)	44.6 (43.1–46.1)	42.0 (39.4–44.7)
At least some college	3,427	8.1	31.7	10.7 (10.3–11.2)	10.3 (9.9–10.7)	44.3 (42.9–45.7)	42.6 (40.5–44.7)
College degree or greater	2,350	5.8	22.9	7.2 (6.8–7.6)	6.7 (6.3–7.0)	36.1 (34.5–37.7)	32.7 (30.4–35.1)
**Employment status**							
Employed/Self-employed	2,716	7.5	29.2	4.8 (4.6–5.1)	5.5 (5.2–5.9)	30.7 (29.4–32.1)	29.8 (28.2–31.4)
Unemployed	215	0.6	2.2	6.6 (5.6–7.8)	8.6 (7.1–10.2)	44.4 (38.8–50.2)	42.4 (36.1–49.0)
Unable to work/ Disabled^§§^	2,904	6.9	26.9	27.6 (26.8–28.4)	26.7 (25.1–28.3)	54.7 (53.5–55.8)	72.2 (69.5–74.7)
Other**^¶¶^**	4,840	10.7	41.6	16.1 (15.5–16.7)	9.4 (8.9–9.9)	43.8 (42.6–45.1)	40.7 (36.4–45.1)
Income-to-poverty ratio***							
Poor/Near poor (<125%)	3,058	6.4	24.9	15.7 (14.8–16.5)	16.4 (15.6–17.1)	63.3 (61.4–65.2)	59.0 (56.1–61.8)
Low income (125% to <200%)	1,855	4.2	16.5	13.4 (12.6–14.2)	12.2 (11.5–13.0)	53.3 (51.0–55.6)	50.9 (47.2–54.7)
Middle income (200% to <400%)	2,962	7.4	28.8	10.4 (9.9–10.9)	9.3 (8.9–9.8)	43.2 (41.7–44.8)	39.3 (36.8–41.8)
High income (≥400%)	2,806	7.7	29.9	7.4 (7.0–7.8)	6.4 (6.1–6.7)	32.8 (31.4–34.2)	28.7 (26.5–30.9)
**Health characteristic**							
**BMI (kg/m^2^)**							
Under/Healthy weight (<25.0)	2,455	5.7	23.2	6.9 (6.5–7.3)	6.8 (6.4–7.1)	39.2 (37.6–41.0)	38.1 (35.2–41.0)
Overweight (25.0 to <30.0)	3,060	7.2	29.4	8.8 (8.4–9.2)	7.4 (7.1–7.8)	39.5 (38.0–41.0)	35.7 (33.3–38.1)
Obese (≥30.0)	4,749	11.7	47.5	16.0 (15.4–16.6)	14.2 (13.7–14.7)	49.8 (48.5–51.2)	45.5 (43.6–47.4)
**Aerobic physical activity level^†††^**							
Meets recommendations	3,073	7.7	30.7	6.0 (5.7–6.4)	6.1 (5.8–6.4)	32.1 (30.9–33.4)	30.8 (29.1–32.5)
Insufficiently active	2,418	5.8	23.1	11.9 (11.2–12.5)	10.2 (9.7–10.8)	43.9 (42.1–45.7)	42.4 (39.5–45.4)
Inactive	4,982	11.6	46.2	17.9 (17.2–18.6)	14.0 (13.4–14.6)	58.0 (56.5–59.4)	54.6 (51.7–57.4)
**Joint symptoms^§§§^**							
Yes	9,401	22.6	88.2	26.9 (26.2–27.6)	21.1 (20.5–21.8)	51.6 (50.5–52.6)	48.8 (47.2–50.4)
No	1,276	3.0	11.8	1.9 (1.7–2.0)	2.0 (1.9–2.1)	20.7 (19.3–22.1)	19.1 (17.2–21.1)
**ADL disability^¶¶¶^**							
Yes	1,236	2.8	10.7	45.3 (42.8–47.7)	34.7 (31.7–37.7)	82.6 (80.2–84.8)	82.7 (75.6–88.1)
No	9,444	22.9	89.3	9.5 (9.2–9.8)	8.7 (8.4–9.0)	41.5 (40.6–42.5)	38.9 (37.6–40.3)
IADL disability****							
Yes	2,476	5.2	20.4	44.9 (43.1–46.6)	34.6 (32.5–36.6)	80.4 (78.6–82.1)	82.4 (78.1–86.0)
No	8,205	20.4	79.6	8.7 (8.4–9.0)	8.0 (7.8–8.3)	39.3 (38.3–40.3)	37.0 (35.6–38.4)
**Psychological distress^††††^**							
None/Mild	5,995	14.2	57.4	7.6 (7.3–7.9)	6.6 (6.3–6.8)	35.5 (34.4–36.5)	30.7 (29.0–32.4)
Moderate	3,122	7.6	30.7	17.8 (16.9–18.6)	17.4 (16.7–18.2)	59.5 (57.7–61.2)	54.1 (51.8–56.5)
Serious	1,213	2.9	11.9	33.8 (31.7–36.0)	31.1 (29.3–32.9)	76.3 (73.5–79.0)	72.3 (68.1–76.1)
**Self-rated health**							
Excellent/Very good	2,290	5.6	21.7	3.7 (3.5–4.0)	3.9 (3.6–4.1)	24.4 (23.2–25.7)	23.3 (21.5–25.2)
Good	3,516	8.4	32.7	12.8 (12.3–13.4)	10.5 (10.1–11.0)	42.9 (41.5–44.3)	39.7 (37.6–41.9)
Fair/Poor	4,868	11.7	45.6	37.1 (35.9–38.3)	29.1 (27.8–30.4)	72.6 (71.1–73.9)	70.2 (67.3–72.9)

**Abbreviations**: AAAL = arthritis-attributable activity limitation; ADL = activities of daily living; AI/AN = American Indian/Alaska Native; API = Asian or Other Pacific Islander; BMI = body mass index; CI = confidence interval; HS = high school; IADL = instrumental activities of daily living; N/A = not applicable; NH = non-Hispanic.

* Age-standardized to the 2000 U.S. projected adult population, using three age groups: 18–44, 45–64, and ≥65 years. Subgroup differences were assessed using pairwise t-tests with significance set at α = 0.05. Results exactly correspond to interpretation of non-overlapping CIs; all categories of education were statistically significantly different from each other per t-test results for unadjusted prevalence of AAAL among all U.S. adults.

^†^ Responded “yes” to, “Are you now limited in any way in any of your usual activities because of arthritis or joint symptoms?”

^§^ Responded “yes” to, “Have you ever been told by a doctor or other health professional that you have some form of arthritis, rheumatoid arthritis, gout, lupus, or fibromyalgia?”

^¶^ Might not sum to overall total for some categories because of item-specific missing data.

** Might not sum to 100 because of rounding.

^††^ Responded “I don't know the answer” to, “Which of the following best represents how you think of yourself?”

^§§^ This category is a combination of respondents self-reporting their reason for not working as: “temporarily unable to work due to health reasons” or “disabled.”

^¶¶^ Students, homemakers, and retirees.

*** Income-to-poverty ratio estimates were derived using NHIS imputed income file. https://www.cdc.gov/nchs/data/nhis/tecdoc18.pdf.

^†††^ Respondents were classified as meets recommendations if they reported ≥150 minutes of moderate intensity leisure time aerobic physical activity per week, insufficiently active if they reported 1–149 minutes, and inactive if they reported 0 minutes. Reported vigorous intensity physical activity minutes were counted twice and added to moderate intensity physical activity minutes.

^§§§^ Responded “yes” to, “The next questions refer to your joints. Please do NOT include the back or neck. During the past 30 days, have you had any symptoms of pain, aching, or stiffness in or around a joint?”

^¶¶¶^ Responded “yes” to, “Because of a physical, mental, or emotional problem, [do you/does anyone in the family] need the help of other persons with personal care needs, such as eating, bathing, dressing, or getting around inside this home?”

**** Responded “yes” to, “Because of a physical, mental, or emotional problem, [do you/any of these family members] need the help of other persons in handling routine needs, such as everyday household chores, doing necessary business, shopping, or getting around for other purposes?”

^††††^ Psychological distress was classified as none/mild, moderate, or severe and measured by the Kessler-6 Scale. https://www.hcp.med.harvard.edu/ncs/k6_scales.php

## Discussion

Annualized estimates from 2016–2018 indicate that the number of U.S. adults with arthritis (58.5 million) and AAAL (25.7 million) increased compared with 2013–2015 estimates (54.4 million and 23.7 million, respectively) (*5*). Arthritis prevalence continues to align closely with projections, but the percentage of the U.S. population reporting AAAL during 2016–2018 (10.4%) had already exactly met the 2020 projection (10.4%) (*4*), continuing a previously observed acceleration in the rise of AAAL (*5*).

Age-standardization had varying effects on subgroup estimates (e.g., changes in magnitude of point estimates [from <1.0 to >10.0 percentage points] and in direction). These shifts reflect both the aging of the U.S. population and that the standard projected 2000 population does not always closely match current demographics for U.S. adults with arthritis, underscoring the importance of focusing on absolute numbers in public health planning. Between the 2013–2015 and 2016–2018 estimates, 4.1 and 2 million more adults reported arthritis and AAAL respectively, continuing a statistically significant linear trend started in 2003–2005 (Figure).

This report characterizes a specific arthritis impact measure, AAAL, and identifies subgroups to prioritize for interventions. The prevalence of both arthritis and AAAL was higher in subgroups representing adults with fewer economic opportunities (i.e., lower education, unable to work or disabled, and lower income-to-poverty ratios), poorer overall health (i.e., higher BMI, less physical activity, more serious psychological distress, and worse self-rated health), and more physical limitations (i.e., joint symptoms in the past 30 days and ADL and IADL disabilities). To address the substantial and growing effects of arthritis and AAAL on the U.S. adult population, it is therefore important to consider adults with this combination of characteristics who would be ideally suited to a multifaceted approach, including intentional outreach to groups at or soon to be at high risk through a social determinants of health approach (*6*), enhanced clinical and community linkages, and more widespread dissemination of evidenced-based public health interventions.

Existing self-management education and physical activity public health interventions that are arthritis-appropriate and inclusive of adults with disabilities have proven benefits, including improved aerobic activity, confidence, and self-rated health and reduced depression, fatigue, and pain (*7*,*8*). These positive effects might be bolstered by combination with medical management, particularly for joint symptoms and mental health. Self-management and clinical efforts might be further enhanced through greater systematic attention to vulnerable groups and by preemptively taking a social determinants of health approach to examine the influence of environment and opportunities on health outcomes, such as for adults whose employment has been negatively affected by arthritis. Persons with rheumatic conditions are known to underuse the Americans with Disabilities Act to address community barriers (e.g., transportation, building access) or receive workplace accommodations, but physician suggestion can increase use, promoting behavior change toward action (*9*). In addition, the Job Accommodation Network^¶¶¶^ is a free service that provides confidential individual counseling, advice, facilitation of job accommodations, and resolution of disability employment issues.

A 2018 study found that symptoms of anxiety are more common than are those of depression among adults with arthritis and more prevalent among these adults aged 18–44 years versus older age groups and in persons with chronic pain versus without (*3*). Psychological distress and despair have previously been identified as contributing factors for excess mortality among all adults aged 25–64 years (*10*). Younger adults with arthritis might especially benefit from mental health screening,**** the functional and psychological benefits of physical activity,^††††^ and clinical interventions for pain and disability management.

The findings in this report are subject to at least two limitations. First, data were self-reported and are subject to recall and social desirability bias. Second, because of the cross-sectional design, a causal relationship between the study outcomes (i.e., arthritis and AAAL) and the characteristics examined cannot be inferred.

During 2016–2018, the estimated number of U.S. adults aged ≥18 years reporting arthritis and AAAL increased by 4.1 and 2 million, respectively, compared with 2013–2015. In addition, AAAL prevalence continues to increase more rapidly than was projected. Because population aging and other contributing factors (e.g., obesity) are expected to sustain these trends, public health, medical, and senior and other service systems face substantial challenges in addressing the needs of adults with arthritis, who already account for nearly one quarter of U.S. adults. A coordinated approach of expanding intervention implementation among adults already limited by arthritis while mitigating future negative arthritis effects by creating “social, physical, and economic environments that promote attaining the full potential for health and well-being,”^§§§§^ could help improve quality of life and limit the personal and societal impacts of arthritis.

**Summary**

**What is already known about this topic?**

Arthritis is a leading cause of disability among U.S. adults. Arthritis‐attributable medical care expenditures and earnings losses were responsible for >$300 billion direct and indirect annual costs in 2013.

**What is added by this report?**

National prevalence of arthritis and arthritis-attributable activity limitations (AAAL) continue to increase in absolute number: 58.5 million (23.7%) U.S. adults have arthritis, 25.7 million (43.9%) of whom have AAAL. Both conditions are most prevalent among adults with worse physical and mental health profiles and more social disadvantage.

**What are the implications for public health practice?**

More widespread dissemination of existing, evidence-based, community-delivered interventions, along with clinical coordination and attention to social determinants of health (e.g., improved social, economic, and mental health opportunities), can help reduce widespread arthritis prevalence and its adverse effects.

## Footnotes

* This category is a combination of respondents self-reporting their reason for not working as: “temporarily unable to work because of health reasons” or “disabled.”

† ADL disability was queried in the Person File and matched to respondents in the Sample Adult file, identified by “yes” to, “Because of a physical, mental, or emotional problem, [do you] need the help of other persons with personal care needs, such as eating, bathing, dressing, or getting around inside this home?”

§ IADL disability was queried in the Person File and matched to respondents in the Sample Adult file, identified by “yes” to, “Because of a physical, mental, or emotional problem, [do you] need the help of other persons in handling routine needs, such as everyday household chores, doing necessary business, shopping, or getting around for other purposes?”

¶ Survey description documents are available at https://www.cdc.gov/nchs/nhis/1997-2018.htm.

** Age-standardized to the 2000 projected U.S. population with three age groups (18–44, 45–64, and ≥65 years). https://www.cdc.gov/nchs/data/statnt/statnt20.pdf

†† Age, sex, race and ethnicity, sexual identity, education, employment status, and income-to-poverty ratio values for the income-to-poverty ratio variable were calculated using NHIS imputed income files https://www.cdc.gov/nchs/data/nhis/tecdoc18.pdf.

§§ Body mass index [weight (kg)/(height [m])^2^] reported as: under/healthy weight (<25.0), overweight (25.0–29.9), or obese (≥30); aerobic physical activity level reported as: active (≥150 minutes), insufficiently active (1–149 minutes), or inactive (0 minutes) moderate-intensity leisure-time aerobic physical activity per 2018 Physical Activity Guidelines for Americans (https://health.gov/sites/default/files/2019-09/Physical_Activity_Guidelines_2nd_edition.pdf); psychological distress (none/mild, moderate, severe measured by the Kessler-6 Scale https://www.hcp.med.harvard.edu/ncs/k6_scales.php); self-rated health (excellent/very good, good, or fair/poor).

¶¶ Measured by joint symptoms (pain, aching, or stiffness in the past 30 days), ADL disability, and IADL disability.

*** https://www.cdc.gov/mmwr/preview/mmwrhtml/mm5540a2.htm?s_cid = mm5540a2_e; https://www.cdc.gov/mmwr/preview/mmwrhtml/mm5939a1.htm?s_cid = mm5939a1_w; https://www.cdc.gov/mmwr/preview/mmwrhtml/mm6244a1.htm; and https://www.cdc.gov/mmwr/volumes/66/wr/mm6609e1.htm

†††
https://surveillance.cancer.gov/help/joinpoint

§§§ 45 C.F.R. part 46.102(l)(2), 21 C.F.R. part 56; 42 U.S.C. Sect. 241(d); 5 U.S.C. Sect. 552a; 44 U.S.C. Sect. 3501 et seq.

¶¶¶
https://askjan.org/about-us/index.cfm

**** https://www.uspreventiveservicestaskforce.org/Page/Document/RecommendationStatementFinal/depression-in-adults-screening1 and https://www.integration.samhsa.gov/clinical-practice/screening-tools#bmb

††††
https://health.gov/paguidelines/guidelines/chapter4.aspx

§§§§
https://health.gov/healthypeople/objectives-and-data/social-determinants-health
